# Single-Cell Transcriptomic Reveals Dual and Multi-Transmitter Use in Neurons Across Metazoans

**DOI:** 10.3389/fnmol.2021.623148

**Published:** 2021-02-01

**Authors:** Clarisse Brunet Avalos, Simon G. Sprecher

**Affiliations:** Department of Biology, University of Fribourg, Fribourg, Switzerland

**Keywords:** neurotransmitter, neurons, scRNAseq, metazoans, Dale's principle

## Abstract

Neurotransmitter expression is widely used as a criterion for classifying neurons. It was initially thought that neurons express a single type of neurotransmitter, a phenomenon commonly recognized as Dale's principle: “one neuron, one transmitter.” Consequently, the expression of a single neurotransmitter should determine stable and distinguishable neuronal characteristics. However, this notion has been largely challenged and increasing evidence accumulates supporting a different scenario: “one neuron, multiple neurotransmitters.” Single-cell transcriptomics provides an additional path to address coexpression of neurotransmitters, by investigating the expression of genes involved in the biosynthesis and transmission of fast-acting neuromodulators. Here, we study neuronal phenotypes based on the expression of neurotransmitters, at single-cell resolution, across different animal species representing distinct clades of the tree of life. We take advantage of several existing scRNAseq datasets and analyze them in light of neurotransmitter plasticity. Our results show that while most neurons appear to predominantly express a single type of neurotransmitter, a substantial number of neurons simultaneously expresses a combination of them, across all animal species analyzed.

## Introduction

Neurons, key components of the nervous system, have been extensively described and characterized. Santiago Ramón y Cajal first introduced the concept of a neuron as a discrete and independent cell within the nervous system, which later laid the foundations for the “neuron doctrine.” Among the elements of this doctrine, Dale's law postulates that a neuron releases a single type of transmitter at all of its synapses, a principle commonly known as “one neuron, one transmitter.” The latter has been widely applied when classifying neurons according to their neurotransmitter phenotype: glutamatergic, GABAergic, cholinergic, glycinergic, or aminergic. Moreover, and in addition to fast-acting transmitters, another layer of complexity is conferred by the expression of neuropeptides, important neuromodulators, extending the list of molecules used for neuronal communication and identification. Nonetheless, neuropeptides are commonly expressed in the company of a given neurotransmitter (Van den Pol, 2012; Nässel, 2018). Recent studies, however, suggest a more complex scenario, where different neurotransmitters coexist in a given neuron, challenging the well-established doctrine when determining cell types.

Dual-transmitter neurons were first reported 40 years ago, and since then different evidences support the coexistence principle. However, most of these works have been done exclusively in chordates, specifically in vertebrates and mammals (Gillespie et al., 2005; Vaaga et al., 2014; Trudeau and El Mestikawy, 2018; Pedroni and Ampatzis, 2019). Since neurons are found widespread across metazoans, from forming relatively simple neuronal networks to complex and centralized brain ganglions, it should be interesting and necessary to probe the validity of Dale's principle and the presence of dual and multi-transmitter neurons among different clades and phyla. Recently, with the advent of single-cell RNA sequencing (scRNAseq), the molecular footprint of individual neurons became accessible. However, the rapid expansion of the technique resulted in an increasing number of available datasets, which commonly remain unexplored and limited to the original analysis. Therefore, further studies represent a powerful source for resolving uncertainties and could potentially change the course of ongoing research toward a more precise and targeted search for knowledge.

**Figure 1:**
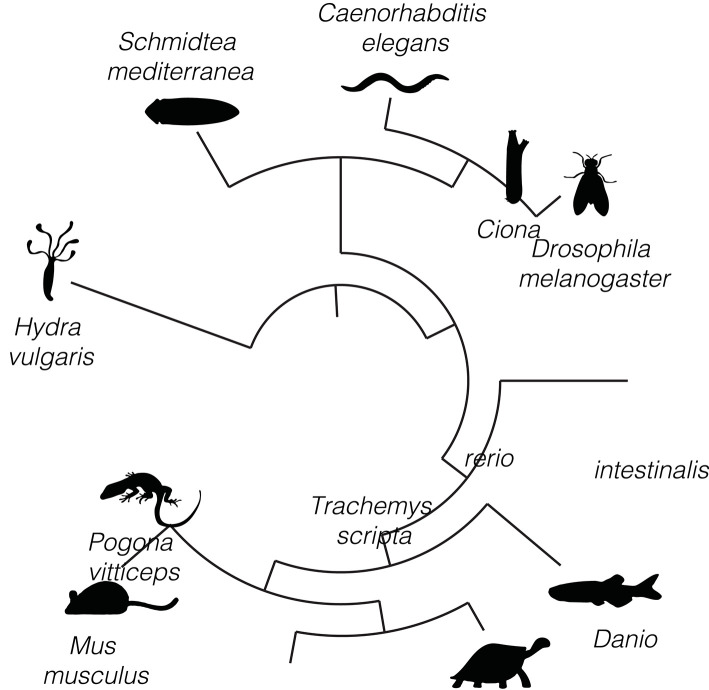
Conserved dual and multi-transmitter neurons across metazoans. **(A)** Circular phylogenetic tree displaying the different species analyzed to study neuronal composition of the brain, or neuronal networks, in terms of their neurotransmitter phenotypes. The species are listed (clockwise) in the order of appearance in the manuscript. Silhouettes are only illustrative.

Here, we provide an extensive evaluation of fast-acting neurotransmitters in neurons populating the brain, or simpler neuronal nets, and its conservation across different species: *Hydra vulgaris* (Siebert et al., 2019)*, Schmidtea mediterranea* (Fincher et al., 2018), *Caenorhabditis elegans* (Cao et al., 2017), *Drosophila melanogaster* (Davie et al., 2018; Brunet Avalos et al., 2019), *Ciona intestinalis* (Sharma et al., 2019), *Danio rerio* (Raj et al., 2018), *Trachemys scripta, Pogona vitticeps* (Tosches et al., 2018), *and Mus musculus* (Ximerakis et al., 2019) (Figure 1). We extended the analysis of existing cell atlases, initially originated by scRNAseq technologies, by the usage of the user-friendly R package Seurat (Butler et al., 2018; Stuart et al., 2019) and assessed whether neurons show an overlapping expression of the canonical marker genes commonly used to define neuronal identity: vesicular transporters and enzymes involved in the biosynthesis and transport of neurotransmitters.

## Materials and Methods

### Data Processing

The R package Seurat (Butler et al., 2018; Stuart et al., 2019) version 3.2.0 was applied to analyze the different datasets. Information about the original datasets is summarized in Table 1. Seurat objects were preprocessed and analyzed following the recommendations of the package developers. Thus, objects were log-normalized with a scale factor of 10,000 to normalize gene expression and undesired sources of variation were removed after applying a linear transformation. Subsequently, variable genes were identified to determine the true dimensionality of the dataset. The number of principal components (PCs) considered for downstream processing was determined with a graph-based approach, Elbow-Plots. Finally, and upon a non-linear dimensional reduction, objects were visually represented in UMAP plots (McInnes et al., 2018). All the parameters applied to the different datasets are summarized in Table 2. Cells expressing a particular gene were determined with the function “WhichCells,” specifying the name of the gene of interest. When multiple genes determined a single cell type, the same procedure was followed, but gene names were specified combining R logical operators. The same applied for coexpressed genes. The results of gene-coexpression analysis were visually represented in Upset plots (Conway et al., 2017).

**Table 1:** Data availability.

**Specie**	**References**	**Identifier**	**Processed data**	**Sampling**
*Hydra vulgaris*	Siebert et al., 2019	GEO: GSE121617	https://singlecell.broadinstitute.org/single_cell/study/SCP260/stem-cell-differentiation-trajectories-in-hydra-resolved-at-single-cell-resolution#study-download	*H. vulgaris* nervous system.
*Schmidtea mediterranea*	Fincher et al., 2018	GEO: GSE111764	GEO: GSE111764	*S. mediterranea* brain.
*Caenorhabditis elegans*	Cao et al., 2017	GEO: GSE98561	–	Whole *C. elegans* cell suspension.
*Drosophila melanogaster* (larva)	Brunet Avalos et al., 2019	GEO: GSE134722	–	*D. melanogaster* larval brain.
*Drosophila melanogaster* (adult)	Davie et al., 2018	GEO: GSE107451	GEO: GSE107451	*D. melanogaster* adult brain.
*Ciona intestinalis*	Sharma et al., 2019	GEO: GSE121807	https://osf.io/ez6rs/	*C. intestinalis* larval brain.
*Danio rerio*	Raj et al., 2018	GEO: GSE105010	GEO: GSE105010	Zebrafish brains (23–25 dpf).
*Trachemys scripta*	Tosches et al., 2018	PRJNA408230	https://public.brain.mpg.de/Laurent/ReptilePallium2018/	*T. scripta* adult pallium.
*Pogona vitticeps*	Tosches et al., 2018	PRJNA408230	https://public.brain.mpg.de/Laurent/ReptilePallium2018/	*P. vitticeps* adult pallium.
*Mus musculus* (Young adult)	Ximerakis et al., 2019	GEO: GSE129788	GEO: GSE129788	Mouse brain (2–3 months) C57BL/6J mice
*Mus musculus* (Old adult)	Ximerakis et al., 2019	GEO: GSE129788	GEO: GSE129788	Mouse brain (21–22 months) C57BL/6J mice

**Table 2:** Parameters applied for the analysis of the different neuronal datasets.

**Neuronal dataset**	**Number of PCs**	**Resolution**	**Orginal/Subcluster**
*Hydra vulgaris*	25	2	Original^*^ (FACS sorted neurons were integrated were non-FACS neurons)
*Schmidtea mediterranea*	25	1	Synaptotagmin > 1
*Caenorhabditis elegans*	40	0.1	Original^*^ (neuronal clustering upon the expression of specific marker genes)
*Drosophila melanogaster* (larva)	38	1	nSyb > 1, elav > 1 and Syt1 > 1
*Drosophila melanogaster* (adult)	40	4	nSyb > 1, elav > 1 and Syt1 > 1
*Ciona intestinalis*	35	0.8	Original^*^ (neuronal clustering upon the expression of specific marker genes)
*Danio rerio*	38	1	gad1b > 1, gad2 > 1, slc32a1 > 1, slc17a6b > 1, slc18a3a > 1, slc44a2 > 1, slc44a5b > 1, slc44a5a > 1 and slc6a9 > 1
*Trachemys scripta*	40	2	Original^*^ (neuronal clustering upon the expression of specific marker genes)
*Pogona vitticeps*	40	2	Original^*^ (neuronal clustering upon the expression of specific marker genes)
*Mus musculus*	30	1	Syt1>1, Slc17a7 > 1, Slc17a6 > 1, Slc17a8 > 1, Gad1 > 1, Gad2 > 1, Slc18a3 > 1, Gm5741 > 1, Slc5a7 > 1, Slc18a2 > 1 and Th > 1

### Code Accessibility

The scripts used for the analysis of the different datasets are accessible at https://github.com/brunetc/Single-cell-transcriptomic-reveals-dual-and-multi-transmitter-use-in-neurons-across-metazoans.git.

## Results

### *Hydra vulgaris* Neurons

**Figure 2:**
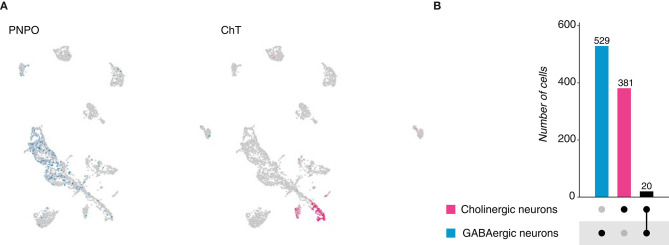
*Hydra vulgaris* neurons. **(A)** UMAP plots showing marker genes expression. *PNPO* labels GABAergic neurons, while *ChT* labels cholinergic ones. PNPO, *pyridoxine-5'-phosphate oxidase and ChT, choline transporter*. **(B)** Upset plot displaying number of cells expressing marker genes used to identified neuronal cell types and their coexpression. Groups are color coded.

The fresh-water polyp, *Hydra vulgaris* belongs to the phylum *Cnidaria*, a sister group of bilaterians. The *Hydra* nervous system is described as a nerve net and can vary from hundreds to thousands of neurons depending on the size of the animal (David, 1973). Given its simplicity, we thought to analyze its cellular composition in terms of neurotransmitter expression, by analyzing the neuronal population from the *Hydra* cell atlas, which was identified upon the expression of a neuronal-specific fluorescent marker and subsequent fluorescence-activated cell sorting (FACS) (Siebert et al., 2019). Two main categories were distinguished: GABAergic and cholinergic neurons. The first group was identified based on the expression of *pyridoxine-5'-phosphate oxidase* (*PNPO*) (Figure 2A), an enzyme responsible for the catalysis of either *pyridoxine 5'-phosphate* (*PNP*) or *pyridoxamine 5'-phosphate* (*PMP*) into *pyridoxal 5'-phosphate* (*PLP*) (Musayev et al., 2003). *PLP* represents the active form of the vitamin B6, which functions as a cofactor in the conversion of glutamate to gamma-aminobutyric acid (GABA). Cholinergic neurons were characterized by the expression of the gene *choline transporter* (*ChT*) (Figure 2A); a protein responsible for the translocation of choline into acetylcholine-synthesizing neurons. We observed that across 3,726 neurons, 529 appeared to be GABAergic and 381 were found to be cholinergic. To determine if *Hydra* neurons escape from Dale's principle, the number of neurons coexpressing *PNPO* and *ChT* was calculated. We distinguished only a small population of 20 cells, which were defined as GABAergic/cholinergic neurons, suggesting neurotransmitter coexpression in the *Hydra* neuronal net (Figure 2B—Supplement Figure 1A). Collectively, these data suggest that in the existing cell atlas of *Hydra vulgaris*, two main neuronal cell types can be distinguished: GABAergic and cholinergic; and that a small portion of them coexpresses marker genes for both cell types. Moreover, a significant number of neurons resulted negative for *PNPO* or *ChT* expression, denoting the presence of additional neuronal types. However, given the incompleteness in the annotation of the *Hydra* genome, their identities remain unknown.

### *Schmidtea mediterranea* Neurons

**Figure 3:**
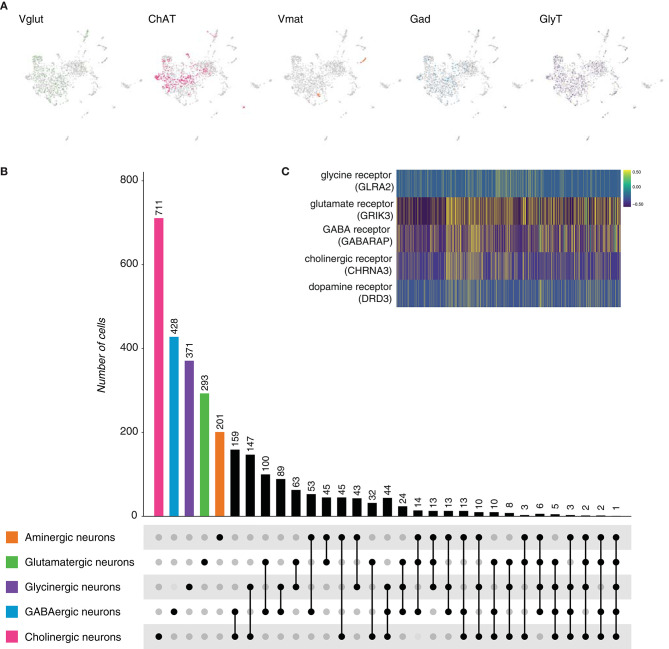
*Schmidtea mediterranea* neurons. **(A)** UMAP plots showing marker gene expression. *VGlut, ChAT, Vmat, Gad*, and *GlyT* label glutamatergic, cholinergic, aminergic, GABAergic and glycinergic neurons, respectively. **(B)** Upset plot displaying the number of cells in each neuronal category. Coexpression was calculated based on the coexpression of above-mentioned marker genes. Groups are color coded. **(C)** Heatmap illustrating the expression and coexpression of some neurotransmitter receptors. GLRA2: *glycine receptor, alpha 2*. GRIK3: *glutamate receptor, ionotropic, kainate 3*. GABARAP: *GABA(A) receptor-associated protein*. CHRNA3: *cholinergic receptor, nicotinic, alpha 3*.DRD3: *dopamine receptor D3*. Each vertical line represents a cell. Expression is color coded.

To verify whether the above observation is also present in its sister group, bilaterians, we started by comparing the planarian *Schmidtea mediterranea*. *S. mediterranea*, as other bilaterians, has a cephalic ganglion, a brain. To better understand its composition, we inspected the neuronal conformation at single-cell scale (Fincher et al., 2018). Out of 7,766 cells, 2,104 were classified as neurons upon the expression of the synaptic marker gene *synaptotagmin* (*Syt, dd_Smed_v4_4222_0_1*). The neuronal population was subclassified according to the expression of genes involved in the biosynthesis, transport or release of neurotransmitters (Table 3), and five main cell types were distinguished: glutamatergic, cholinergic, aminergic, GABAergic, and glycinergic neurons (Figure 3A). Acetylcholine appeared to be the main neurotransmitter in the brain of *S. mediterranea*, followed by GABA, glycine, glutamate, and the different monoamines. Coexpression of the marker genes for each neuronal subclass was assessed and computed. Coexpression was considered positive when one of the marker genes of each neuronal subclass was coexpressed with that of a second subclass (Supplement Figure 1B). A subset of cells showed to be dual-transmitter neurons, where most of them express acetylcholine in combination with a second neurotransmitter. Additionally, we observed a small subset of neurons expressing marker genes for more than two types of neurotransmitters (Figure 3B). Given the broad combinatorial code in the planarian neurons, we thought to investigate the expression of the receptors for the above-mentioned neurotransmitters. We observed that in many cases, these receptors were coexpressed suggesting that neurons are prone to respond to different neurotransmitters by making use of different receptors (Figure 3C). Collectively, these results indicate that five neuronal types can be distinguished from the existing cell atlas of *S. mediterranea* brain. In addition, multiple subsets of neurons showed to coexpress more than a single type of neurotransmitter. However, the number of neurons decreases with the increase of types of neurotransmitters coexpressed.

**Table 3:** Marker genes used to classify the neuronal set from *Schmidtea mediterranea*.

**Neuronal cell type**	**Marker genes**
Aminergic neurons	*Vmat (dd_Smed_v4_19744_0_1)* *SerT (dd_Smed_v4_12700_0_1)* *Ddc (dd_Smed_v4_21108_0_1)* *Tph (dd_Smed_v4_8392_0_1)* *Th (dd_Smed_v4_16581_0_1)*
Cholinergic neurons	*Chat (dd_Smed_v4_11968_0_1)* *Chat (dd_Smed_v4_6208_0_1)*
GABAergic neurons	*Gad (dd_Smed_v4_12653_0_1)* *TauT (Tomi et al., 2008)* *(dd_Smed_v4_6616_0_19)* *GAT (dd_Smed_v4_11826_0_1)*
Glutamatergic neurons	*Vglut (dd_Smed_v4_10192_0_1)*
Glycinergic neurons	*GlyT (dd_Smed_v4_5713_0_1)*

### *Caenorhabditis elegans* Neurons

**Figure 4:**
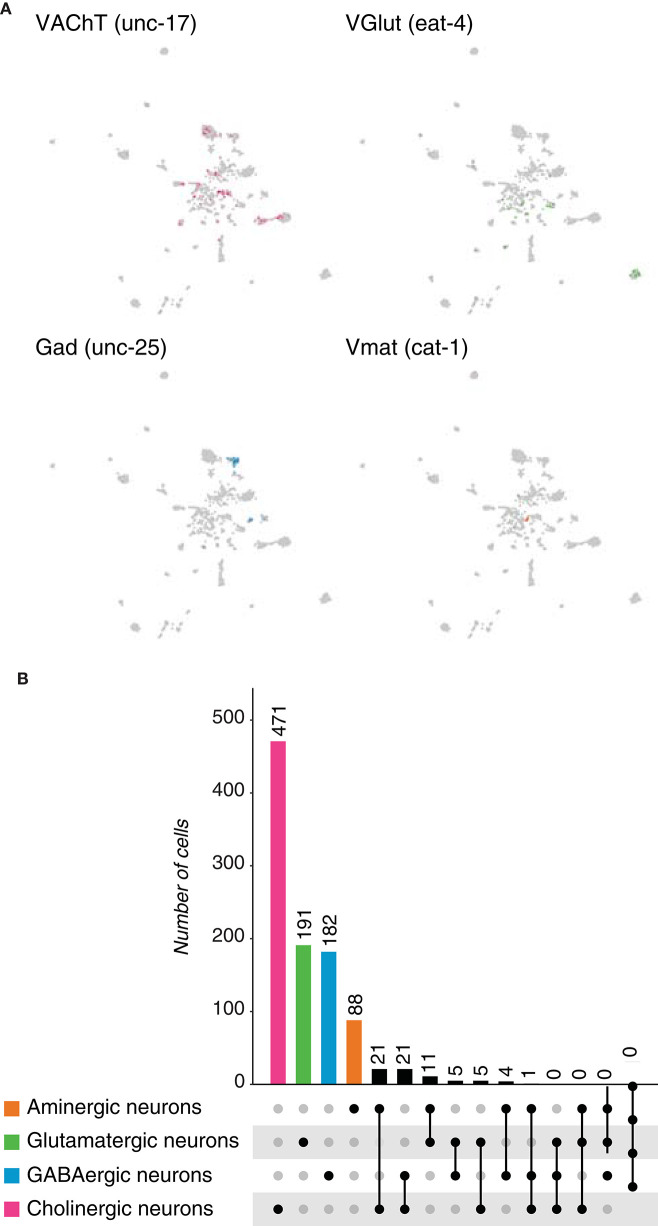
*Caenorhabditis elegans* neurons. **(A)** Expression of marker genes used to identify neurons based on their neurotransmitter phenotype represented in UMAP plots. *VAChT, VGlut, Gad* and *Vmat* label cholinergic, glutamatergic, GABAergic and aminergic neurons, respectively. **(B)** Upset plot displaying number of cells in each neuronal category. Coexpression was calculated based on the coexpression of above-mentioned marker genes. Groups are color coded.

Next we thought to investigate whether neurotransmitter coexpressing neurons could be also distinguished in the nematode *Caenorhabditis elegans* (*C. elegans*) cell atlas (Cao et al., 2017). We analyzed the original neuronal population, identified based on the expression of neuronal genes: *carboxypeptidase E* (*egl-21*), *neuroendocrine convertase 2 (egl-3*) and *receptor-type tyrosine-protein phosphatase-like ida-1* (*ida-1*), among others; and identified neurotransmitter phenotypes upon the expression of marker genes (Table 4, Figure 4A). In accordance to recent studies (Serrano-Saiz et al., 2017), acetylcholine and GABA were the most and least abundant neurotransmitters, respectively. Glutamatergic neurons represented the second most abundant type of neurons in the nematode, after GABAergic ones. In addition, monoamines were found to be expressed in a minority subset of neurons; where serotonergic, dopaminergic and tyraminergic/octopaminergic neurons were distinguished, as they expressed *tryptophan hydroxylase (TPH), tyrosine hydroxylase* (*TH*) and *tyrosine decarboxylase (TDC)*, correspondingly (Supplementary Figure 2). For comparison purposes, all monoaminergic neurons were later grouped in a single category. We next quantified the number of dual or multi-transmitter neurons. Out of the 2,301 neurons detected in the scRNAseq dataset, ~1% coexpresses acetylcholine and an aminergic transmitter, or acetylcholine and GABA (Figure 4B, Supplementary Figure 1C). All other dual combinations were shown to be possible, but at a lower frequency. Moreover, we identified a single neuron expressing marker genes for more than two different neurotransmitters. Collectively, these results illustrate the main neuronal types present in the brain of *C. elegans*: cholinergic, glutamatergic, GABAergic and aminergic neurons; and shows that our single-cell analysis results are in accordance to previous reports. Furthermore, dual-transmitter neurons are also present in *C. elegans*, but multi-transmitter neurons seem to be a rare event in this particular nervous system.

**Table 4:** Marker genes used to classify the neuronal set from *C. elegans*.

**Neuronal cell type**	**Marker genes**
Aminergic neurons	*Vmat (cat-1)* *Tph (tph-1)* *Ddc (bas-1)* *SerT (mod-5)* *Th (cat-2)* *Dat (dat-1)* *Tdc (tdc-1)* *Tbh (tbh-1)*
Cholinergic neurons	*Chat (cha-1)* *VAChT (unc-17)* *ChT (cho-1)*
GABAergic neurons	*Gad (unc-25)* *VGAT (unc-47)* *GAT (snf-11)*
Glutamatergic neurons	*Vglut (eat-4)*

### *Drosophila melanogaster* Neurons

**Figure 5:**
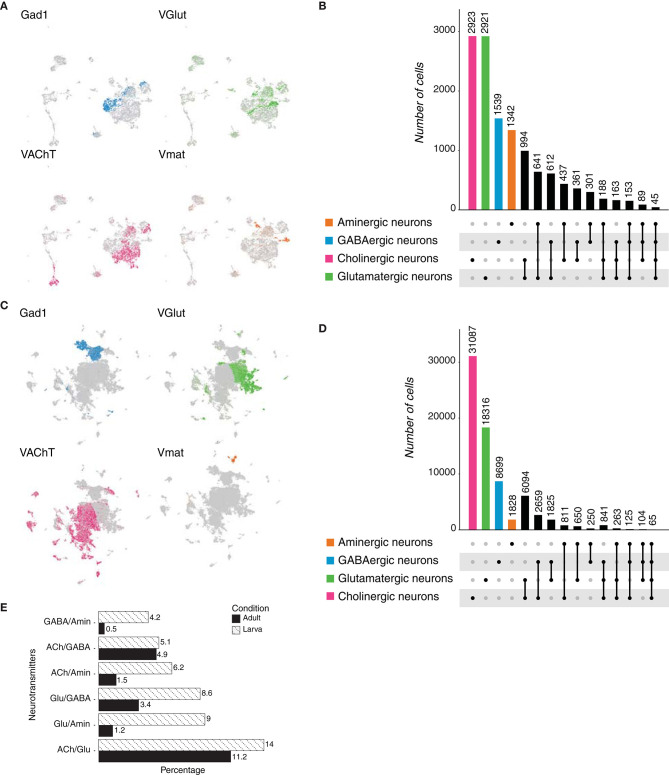
*Drosophila melanogaster* neurons. **(A)** Expression of marker genes used to identify neurons based on their neurotransmitter phenotype in the *Drosophila* larval brain represented in UMAP plots. Gad1, *VGlut, VAChT* and *Vmat* label GABAergic, glutamatergic, cholinergic and aminergic neurons, respectively. **(B)** Upset plot displaying number of cells in each neuronal category. Neurotransmitter coexpression was calculated based on the overlaps of marker genes mentioned above. Groups are color coded. **(C)** Expression of the above-mentioned marker genes in the adult brain of *D. melanogaster* represented in UMAP plots. **(D)** Upset plot displaying number of cells in each neuronal category. Coexpression was calculated based on the coexpression of above-mentioned marker genes. Groups are color coded. **(E)** Dual-transmitter neurons are less frequent in the adult brain in comparison to the larval one. Cell numbers are represented as percentages calculated from the total of neurons for each dataset.

We next focused on the arthropod *Drosophila melanogaster*. First, neurons were identified from the larval brain cell atlas (Brunet Avalos et al., 2019) based on the expression of the pan-neuronal marker *embryonic lethal abnormal vision* (*elav)* and the synaptic markers: *synaptotagmin 1* (*Syt1*) and *neuronal synaptobrevin* (*nSyb*). The 7,102 neuronal cells were classified as cholinergic, glutamatergic, GABAergic or aminergic, as they expressed: *vesicular acetylcholine transporter* (*VAChT*), *vesicular glutamate transporter* (*VGlut*), *glutamic acid decarboxylase 1* (*Gad1*) or *vesicular monoamine transporter* (*Vmat*), respectively (Figure 5A). Acetylcholine and glutamate were found to be the most frequently expressed neurotransmitters in the larval brain, followed by GABA and then by the monoamines. We then quantified the overlaps among the above-mentioned marker genes, and observed coexpression for all possible combinations (Figure 5B). In *Drosophila*, GABA is found in inhibitory synapses, while glutamate and acetylcholine are majorly present in excitatory ones (Wilson and Laurent, 2005; Liu and Wilson, 2013). Surprisingly, 612 cells expressed *Gad1* and *VGlut* simultaneously, while 361 cells exhibited coexpression of *Gad1* and *VAChT*; suggesting that a single neuron could potentially displayed excitatory and inhibitory behaviors. In addition, few cells showed to coexpress three or more fast-acting neurotransmitters.

We thought to investigate whether the observations we made in the larval brain, also occurred in the context of the adult brain. For this, we analyzed the existing single-cell catalog (Davie et al., 2018) and identified neuronal populations as described above (Figure 5C). We found that similar to the larval brain, acetylcholine and glutamate were the most abundant neurotransmitters; but, surprisingly, the difference among their levels of expression became larger in the adult brain. Thus, the large majority of neurons in the adult brain showed to be cholinergic. We then estimated the frequency of dual or multi-transmitter neurons and observed that, as expected, acetylcholine and glutamate represented the most frequent combination (Figure 5D, Supplementary Figure 1D). In addition, we observed that, with the exception of cholinergic/GABAergic neurons, dual-transmitter neurons were less frequent in the adult brain than in the larval one, probably indicating that neurons tune their behavior during development (Figure 5E). Moreover, most dual-transmitter neurons in the larval brain express glutamate, while in the adult brain acetylcholine was found in most dual-transmitter neurons. Furthermore, the number of multi-transmitter neurons was inversely proportional to the number of expressed neurotransmitters. Collectively, these data indicate that in *D. melanogaster* larval brain the most abundant neurotransmitters are acetylcholine and glutamate, while in adult neurons acetylcholine becomes the most frequent one. In addition, a small but significant number of neurons were shown to coexpresses more than a single type of fast-acting neuromodulators.

### *Ciona intestinalis* Neurons

**Figure 6:**
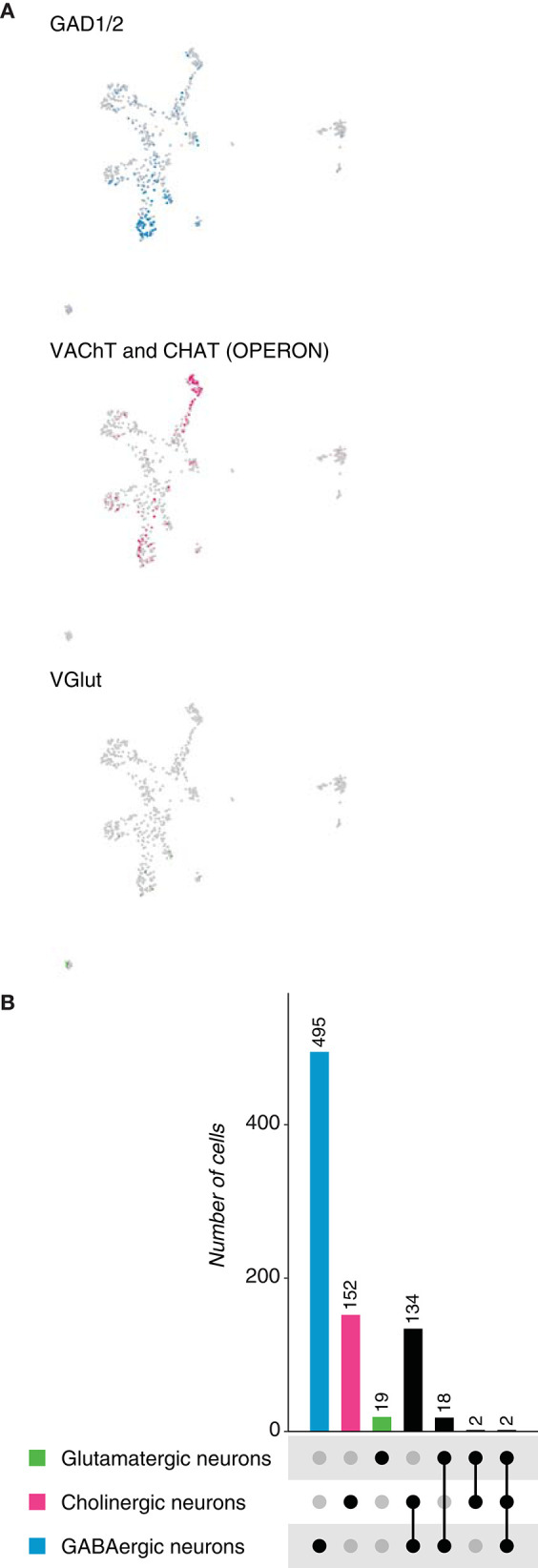
*Ciona intestinalis* neurons. **(A)** Expression of marker genes used to identify neurons based on their neurotransmitter phenotype represented in UMAP plots. Gad1/2, *VAChT* and *CHAT*, and *VGlut* label GABAergic, cholinergic and glutamatergic neurons, respectively. **(B)** Neuronal quantification based on the expression of the above-mentioned marker genes shows neurotransmitter coexpression. Upset plot illustrates number of cells for each neuronal category. Groups are color coded.

To further probe Dale's principle in chordates, we first analyzed the neuronal population of the tunicate *Ciona intestinalis* larval brain, identified upon the expression of the neuronal markers *Synaptotagmin 1* (*Syt1*), *CUGBP Elav-like family member 3/4/5* (*Celf3/4/5*), *retinaldehyde-binding protein 1* (*Rlbp1*), among others (Sharma et al., 2019). Neurotransmitter expression was assessed and three main cell types were distinguished: GABAergic neurons (*GAD1/2, glutamate decarboxylase 1/2 and VGAT, vesicular GABA transporter*), cholinergic neurons (*VAChT, vesicular acetylcholine transporter and CHAT, acetylcholine transporter*) and glutamatergic neurons (*VGlut, vesicular glutamate transporter*) (Figure 6A). The neuronal dataset comprised 644 cells, in which GABA appeared to be the most frequently expressed neurotransmitter (495 cells), followed by acetylcholine (152 cells) and glutamate (19 cells) (Figure 6B). These results indicate that in contrast to the previously analyzed bilaterians, but similarly to *Hydra vulgaris*, GABA is the main neurotransmitter in tunicates. Surprisingly, we did not observe the expression of *tyrosine hydroxylase* in the neuronal object, which has been reported to be expressed in the so called coronet cells, previously reported to be immunoreactive to dopamine (Dilly, 1969). We next examined overlapping expression of the above-mentioned marker genes. Remarkably, 134 neurons showed coexpression of marker genes for GABA and acetylcholine (Supplementary Figure 1E), while 18 were dual-transmitter neurons for GABA and glutamate; indicating that almost all glutamatergic neurons coexpress GABA. In addition, two neurons coexpress acetylcholine and glutamate. Thus, expectedly, only two neurons appeared to express GABA, glutamate and acetylcholine, simultaneously. Collectively, these data show that the main neurotransmitter in neurons of *Ciona intestinalis* is GABA, and that a relatively large number of these neurons coexpress a second fast-acting transmitter. Moreover, all glutamatergic neurons seemed to be dual-transmitter neurons, with GABA or acetylcholine as the second neurotransmitter.

### *Danio rerio* Neurons

**Figure 7:**
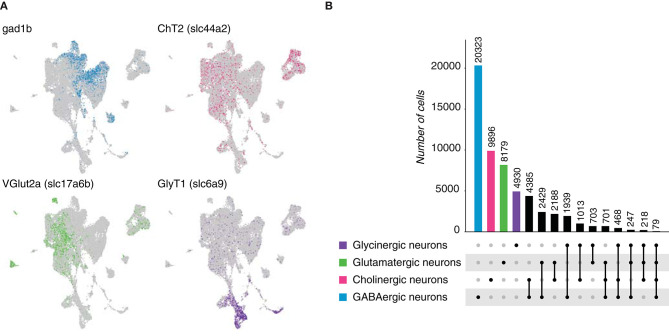
*Danio rerio* neurons. **(A)** Zebrafish neurons can be classified based on the expression of marker genes involved in the biosynthesis and transmission of neurotransmitters. UMAP plot showing the expression of Gad1b, *ChT2, VGlut* 2a and *GlyT1*, which label GABAergic, cholinergic, glutamatergic and glycinergic neurons, respectively. **(B)** Neuronal quantification based on the expression of the above-mentioned marker genes shows neurotransmitter coexpression in the zebrafish brain. Upset plot illustrates number of cells for each neuronal category. Groups are color coded.

Vertebrates account for the vast majority of chordates, hence we analyzed the neuronal population of the brain of *Danio rerio*, commonly known as zebrafish, at single-cell resolution (Raj et al., 2018). A neuronal data set was built following the expression of genes involved in the synthesis or transport of neurotransmitters (Table 5, Figure 7A) and consisted of 32,226 cells. We found that GABA was the most frequently expressed neurotransmitter, followed by acetylcholine and glutamate. In addition, we could also distinguish a population of glycinergic neurons in contrast to the previously-analyzed species. Dual-transmitter neurons in the zebrafish central nervous system have been previously explored, but covering only the spinal cord (Pedroni and Ampatzis, 2019). With the multi-transmitter neurons phenomenon unexplored in the brain, we decided to quantify the number of cells displaying dual or multi-expression of neurotransmitters. Coexpression of marker genes for two different neurotransmitters was observed for all possible combinations in large subset of cells (Figure 7B, Supplementary Figure 1F), indicating that dual-transmitter neurons is common phenomenon in the zebrafish brain. Furthermore, dual-transmitter neurons showed expression of marker genes for excitatory and inhibitory modulators, demonstrating that Dale's principle is a limited approach to classify neurons. Moreover, and as observed in the spinal cord (Pedroni and Ampatzis, 2019), a small population of neurons displayed triple and even quadruple coexpression of marker genes for different neurotransmitters. Together, these data provide evidence of multi-transmitter neurons in the zebrafish brain, pointing that neurons cannot be simply classified based on the expression of a particular neurotransmitter.

**Table 5:** Marker genes used to classify the neuronal set from *D. melanogaster*.

**Neuronal cell type**	**Marker genes**
Cholinergic neurons	*slc18a3a (VAChT)* *slc44a2 (ChT2)* *slc44a5a (ChTl5a)* *slc44a5b (ChTl5b)*
GABAergic neurons	*gad1b* *gad2* *slc32a1 (VGAT)*
Glutamatergic neurons	*slc17a6b (VGlut 2.1)*
Glycinergic neurons	*slc6a9 (GLYT1)*

### Dual-Transmitter Neurons in Reptiles

**Figure 8:**
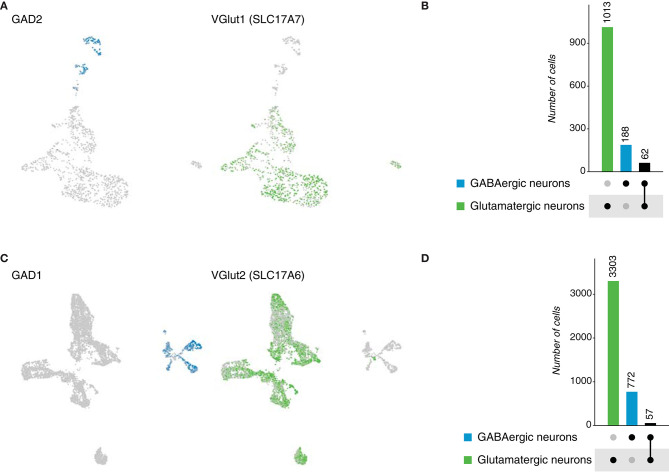
Reptile neurons. **(A)** UMAP plots showing the expression of marker genes used to classify neurons according to their neurotransmitter phenotype in the lizard brain. *Gad2* and *VGlut1* label GABAergic and glutamatergic neurons, respectively. **(B)** Neuronal quantification based on the expression of the above-mentioned marker genes shows neurotransmitter coexpression in the lizard brain. Upset plot illustrates number of cells for each neuronal category. Groups are color coded. **(C)** UMAP plots showing the expression of marker genes used to classify neurons according to their neurotransmitter phenotype in the turtle brain. *Gad2* and *VGlut1* label GABAergic and glutamatergic neurons, respectively. **(D)** Neuronal quantification based on the expression of the above-mentioned marker genes shows neurotransmitter coexpression in the turtle brain. Upset plot illustrates number of cells for each neuronal category. Groups are color coded.

To expand our understanding of neurotransmitter expression in the brain of chordates, we analyzed single-cell data sets of reptiles: *Pogona vitticpes* (lizard) and *Trachemys scripta* (turtle). For both species, the original neuronal datasets, classified based on the expression of neuronal marker genes (Tosches et al., 2018), were inspected to determine the expression of neurotransmitters. In the case of the lizard, 1,160 neurons were analyzed and later classified as glutamatergic (*SCL17A7*, 1,013 cells) and GABAergic (*GAD2*, 188 cells) (Figures 8A,B). For the turtle, 5,901 neurons were analyzed; from which 3,303 and 772 neurons were classified as glutamatergic and GABAergic, respectively (Figures 8C,D). No other neurotransmitters were identified, not even in the original dataset containing other cell types, but evidence supports the existence of cholinergic neurons in reptiles (Brauth et al., 1985; Powers and Reiner, 1993). However, the inspected datasets focused only on the dorsal telencephalon. Next, we investigated the existence of dual-transmitter neurons in both species. A similar number of glutamatergic/GABAergic neurons was obtained in both cases, with 62 and 57 neurons in the lizard and turtle brains, correspondingly (Supplementary Figure 1G). Together, these data provide evidence of dual-transmitter neurons in reptiles for the first time, indicating that glutamate and GABA could potentially coexist in some synapses, challenging the current system applied to classify neurons. as excitatory or inhibitory.

### *Mus musculus* Neurons

**Figure 9:**
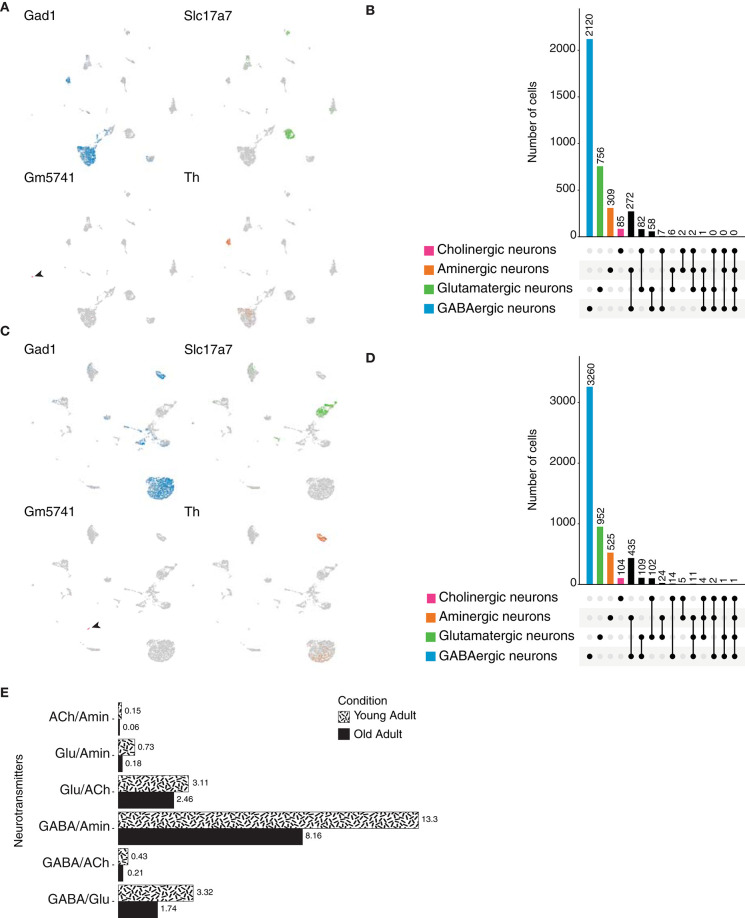
*Mus musculus* neurons. **(A)** UMAP plots showing the expression of marker genes used to classify neurons according to their neurotransmitter phenotype in the young adult mouse brain. *Gad1, Slc17a7* (*VGlut1), Gm5741* and *Th* label GABAergic, glutamatergic, cholinergic and aminergic neurons, respectively. **(B)** Cell quantification based on the expression of the above-mentioned marker genes shows neurotransmitter coexpression in the young adult mouse brain. Upset plot illustrates number of cells for each neuronal category. Groups are color coded. **(C)** Expression of the above-mentioned marker genes in the aging mouse brain represented in UMAP plots. **(D)** Upset plot displaying number of cells in each neuronal category for the aging mouse brain. Coexpression was calculated based on the coexpression of above-mentioned marker genes. Groups are color coded. **(E)** Dual-transmitter neurons comparison between the young and aging mouse brains indicate that there is no substantial change in terms of dual-transmitter neurons composition, with the sole exception of GABAergic/aminergic neurons that seem to be increase with aging. Cell numbers are represented as percentages calculated from the total of neurons for each dataset.

We next focused on mammals, more specifically on the rodent *Mus musculus*. We analyzed the published atlas of the mouse adult brain (Ximerakis et al., 2019) and focused on the neuronal population identified based on the expression of genes involved in the biosynthesis or release of neurotransmitters. We identified GABAergic, glutamatergic and cholinergic neurons, as they express *Gad1* or *Gad2* (*glutamic acid decarboxylase 2*); *VGlut1 (vesicular glutamate transporter 1), VGlut2 (vesicular glutamate transporter 2)* or *VGlut3 (vesicular glutamate transporter 3)*; and *VAChT, Gm5741 [guanine nucleotide-binding protein subunit gamma*, previously identified to be expressed in cholinergic neurons by Ximerakis et al. (2019)] or *Cht1 (choline transporter 1)* and *Glyt2 (glycine transporter 2)*, respectively. Aminergic neurons were classified based on the expression of *Vmat2 (Synaptic vesicular amine transporter)* or *Th (Tyrosine 3-monooxygenase)*, as their expression did not perfectly overlap (Figure 9A). Out of 3,332 cells, marker genes for GABA transmission represented the most frequently expressed ones, found in 2,120 neurons; suggesting the predominant use of GABA in mouse neurons (Figure 9B). Glutamatergic and aminergic neurons occupied the second and third places, respectively. Surprisingly, only a small fraction of cells expressed marker genes involved in the transport of acetylcholine, resulting in a small population of cholinergic neurons. These results indicate that similarly to the neurons of *Danio rerio*, and conversely to the scenario presented in the brain of *Drosophila*, the vast majority of the mouse neurons are GABAergic.

Dale's principle has been extensively characterized in chordates, in particular in mammals. However, the techniques used then to addressed this phenomenon lack the transcriptional component. Thus, we thought to validate those observations at a transcriptional level in the context of the mouse adult brain. Remarkably, coexistence of markers genes for aminergic neurons and *Gad1* or *Gad2*, appeared as the most common case of dual-transmitter neurons (Supplementary Figure 1H). Moreover, a group of neurons displayed expression of different vesicular glutamate transporters and *Gad1* or *Gad2*; showing that a given neuron in the mouse brain can display both excitatory and inhibitory behaviors. We then thought to investigate triple-transmitter neurons, and as in the case of the species analyzed above, the number of neurons decreased as the number of co-expressed neurotransmitters increased.

Next, to address whether neurotransmitter phenotype in the mouse brain is a dynamic one, we analyzed and compared the mouse aging brain. The different neuronal populations were identified as described above (Figure 9C), and similarly to the young adult brain, upon quantification, GABAergic neurons were shown to be the most frequent neurons in the aging brain (Figure 9D). In line with these results, most dual-transmitter neurons express *Gad1* or *Gad2* with a monoamine or glutamate. We observed a slight increase in the number of multi-transmitter neurons in comparison to the young adult brain, likely due to the fact that the aging dataset comprises a larger number of cells. Nevertheless, the comparison of dual-transmitter neurons across life stages did not yield significant differences, suggesting that throughout adulthood and aging, once established, these neurons remained more or less invariable, with the exception of GABAergic/monoaminergic neurons, which were shown to increase with age (Figure 9E). Collectively, these results illustrate the complexity of cell-type determination due to the plasticity found in neurons in terms of neurotransmitter expression. We extended our previous findings to the mammalian brain, where single-transmitter neurons coexist with a large pool of dual-transmitter ones, and with a much lower number of triple or quadruple-transmitter neurons. Furthermore, we showed that through adulthood and aging dual-transmitter neurons do not suffer significant changes, suggesting that once established, these neurons maintain their fates and functions.

## Discussion

In the present study, we present an extensive characterization of the neuronal phenotype in relation to the expression of neurotransmitters among different species of metazoa. Single-cell sequencing technology has revolutionized the way in which cells are studied, providing valuable information about the cellular organization in terms of its transcriptional composition. However, with the rapid expansion of the technique, incredibly large amounts of cells are daily sequenced, generating a growing number of available cell-type and tissue-specific transcriptome libraries. Commonly, the study of these libraries is reduced to the original analysis, while many pathways remained unexplored. We here reanalyzed existing brain or neuronal scRNAseq datasets and inspected their composition based on the expression of canonical marker genes. This initial comparison of the neuronal composition, in terms of its neurotransmitter content, provides evidence about the existence of dual or multi-transmitter neurons in different clades of the tree of life.

The neurotransmitter phenotype was once thought to be a static characteristic. However, Dale's principle has been repeatedly challenged toward a more plastic and versatile conception, since Tomas Hökfelt introduced the coexistence law 40 years ago. Nevertheless, the evidence accumulated supports cotransmitter neurons, almost uniquely, in chordates, where most experiments focused on the mammal nervous system, leaving aside many other species. Thus, the evolution of the nervous system can provide clues about the origin and conservation of dual or multi-transmitter neurons. Early branching species, such as the cnidarian *Hydra vulgaris*, which possess a relatively simple neuronal net can shed new light into this topic. In the present study, we report the coexistence of a small set of GABAergic/cholinergic neurons, which presumably indicates that this type of neurons were present at the origin of the nervous system. It will be interesting to continue studying the hydra neurons, as their genome continues to be annotated, as it can provide clues about the existence of other neuromodulators which today cannot be identified. Following an evolutionary perspective, we propose that transmitter plasticity observed in neurons across different species of animals constitutes a conserved phenomenon, probably providing an efficient way to refine neuronal networks during development as well as enabling fast responses to certain stimuli in the adult brain. In many neurons fast-acting neurotransmitters are accompanied by other neuromodulators, such as neuropeptides, increasing the complexity of neuronal classification. In addition, the difference in the number of cells coexpressing different neurotransmitters within the larval and adult brain of *D. melanogaster*, suggest a dynamic process for neurotransmitter specification. We hypothesized that neurons in the fly brain tune their behavior during development, similarly to what has been observed in developing auditory system of rats (Gillespie et al., 2005). Conversely, the ratio of dual-transmitter neuron remained almost invariable through aging in the mouse brain, suggesting that once the cell fate has been established these neuronal types seem to not be particular sensitive to aging. It would be interesting to analyze how this ratio changes from the embryo to the adult brain; however, clever experimental design is needed to have comparable datasets at different developmental time points.

We here investigated the validity of Dale's principle across different species, for which antibodies or genetic manipulations are not readily accessible, but scRNAseq became available, to illustrate the diversity and conservation of neurotransmitter phenotype. Our results, further support the observations made for the nematode *C. elegans*, where the interneurons PVN and PNN were shown to cotransmit GABA and acetylcholine (Serrano-Saiz et al., 2017), among other cases of cotransmitter neurons. This phenomenon has also been recently addressed in arthropods, more precisely in *D. melanogaster*, where different tools for genetic manipulation of neuronal expression exist. However, most of these studies focused on the coexpression of a neuropeptide with a fast-acting neurotransmitter, as it was described for the circadian clock neurons (Choi et al., 2012) and extensively revised by Nässel (2018). However, the possible existence of dual-transmitter neurons *per se* was only highlighted upon the first scRNAseq studies in the brain (Croset et al., 2018); but, these neurons need to be further characterized to better understand their function and the regulation behind neurotransmitter plasticity. Analogously, cotransmitter neurons have been reported in the zebrafish spinal cord, where ~15% of the neurons were shown to exhibit a plastic neurotransmitter phenotype (Pedroni and Ampatzis, 2019). Here we presented a similar scenario for the brain, where most dual-transmitter neurons express GABA in combination with a second transmitter; furthermore, a neuronal subset showed to display a more laxed transmitter phenotype, with the expression of marker genes for more than two neurotransmitter types. Moreover, neurons in the mammalian brain were shown to coexpress transmitters with opposite functions, as it is the case of glutamate and GABA (Beltrán and Gutiérrez, 2012; Choi et al., 2012; Root et al., 2014; Yoo et al., 2016; Galván and Gutiérrez, 2017), which are known to display excitatory and inhibitory behaviors, respectively. In the auditory system, the presence of *Vglut3* in GABA/glycinergic neurons propose that glutamate cotransmission plays a key role in synaptic reorganization and the refinement of the inhibitory circuit (Noh et al., 2010). These observations are consistent with our findings, where glutamatergic/GABAergic neurons constituted one of the most frequent cases of dual-transmitter neurons in the mouse brain. In addition, we found a large number of neurons coexpressing monoamines with glutamate or GABA, in accordance with previous reports where TH positively labeled glutamatergic neurons in the mouse and rat brains (Fung et al., 1994). Similarly, we found a large population of glutamatergic/GABAergic neurons in the *Drosophila* brain, which also suggests the coexistence of neurotransmitters with opposite behaviors. However, careful inspection is needed knowing that in invertebrates and in particular in *D. melanogaster* glutamate can serve as an inhibitory or excitatory neurotransmitter depending on the neuronal type (Daniels et al., 2004; Liu and Wilson, 2013; Lacin et al., 2019), suggesting that the neuronal coding is indeed more complex than first expected. Equivalently, in *C. elegans* glutamate has been shown to be able to act as an inhibitory neurotransmitter in the olfactory circuit (Chalasani et al., 2007), enabling a tighter network refinement.

The principle of “one neuron, one transmitter” has been extensively applied for neuronal identity determination in scRNAseq experiments. Based on the expression of canonical marker genes, such as vesicular transporters or enzymes involved in the biosynthesis of neurotransmitters, neurons are generally classified as cholinergic, glutamatergic, GABAergic, glycinergic or aminergic. Meticulous inspection of neuronal transcriptomes revealed coexistence of these markers in subsets of neurons, indicating that a complementary strategy is necessary to bona fide determine cell types. In particular, much more benefit can be gained from the state-of-the-art techniques available today. Combinatorial approaches, which enable the identification of cell type-specific transcriptional factors and with this the characterization of core regulatory complexes (CoRCs) for each particular cell type, can provide additional validation when determining neuronal identities (Arendt et al., 2016). Moreover, single-cell sequencing together with machine-learning algorithms can predict potential differentiation factors to later accelerate high-throughput screenings of adult differentiated cell types, as the case of neuronal subtypes (Konstantinides and Desplan, 2020). Furthermore, cell type-specific transcriptional regulators may have different roles or functions depending on the genomic landscapes; therefore, single-cell epigenomics can provide an attractive opportunity to unravel the molecular details determining cell fates (Shema et al., 2019). Additionally, novel approaches such as Axon-seq (Nijssen et al., 2018), which guarantee access to the axonal microenvironment, often lost during sampling preparation for scRNAseq, constitute a valuable source to further investigate synaptic translation (Holt and Schuman, 2013) and plasticity at the transcriptional level. Hence, much insight can be gained with a combinatorial approach, which always has to be accompanied by an ingenious experimental design that allows data to be obtained at different time points and by different technologies.

Here we provided an extensive characterization of the neuronal transcriptome in terms of its neurotransmitter content. By applying a well-established and popular method to analyze scRNAseq results, we used existing datasets to answer unsolved questions, an approach that could be easily extended to other biological interrogations. Although our findings further support a theory that has been around since the possibility of neurons releasing more than a single neurotransmitter was first considered (Burnstock, 1976), many aspects need to be addressed. Finally, it would be interesting to understand the role that cotransmission plays in different neuronal networks, how this process is regulated during development and the implication that this phenotype could have in the context of an injury or disease.

## Data Availability Statement

The original contributions presented in the study are publicly available. This data can be found here: https://github.com/brunetc/Single-cell-transcriptomic-reveals-dual-and-multi-transmitter-use-in-neurons-across-metazoans.git.

## Author Contributions

CBA performed data analysis and wrote the manuscript. SS conceived the study, participated in the design of the study and helped draft the manuscript.

## Conflict of Interest

The authors declare that the research was conducted in the absence of any commercial or financial relationships that could be construed as a potential conflict of interest.

## Supplementary Material

Supplementary Figure 1 Coexpression analysis. UMAP plots illustrating the coexpression of marker genes used to subclassify neurons based on their neurotransmitter phenotypes. **(A)** Neurons of *Hydra vulgaris* coexpressing *PNPO* (red) and *ChT* (green) result in GABAergic/Cholinergic dual-transmitter neurons (yellow). **(B)** Neurons of *Schmidtea mediterranea* coexpressing *ChAT* (red) and *Gad* (green) result in Cholinergic/GABAergic dual-transmitter neurons (orange). **(C)** Neurons *of Caenorhabditis elegans* coexpressing *VAChT* (red) and *Gad1* (green) result in Cholinergic/GABAergic dual-transmitter neurons (yellow/orange). **(D)** Neurons *of Drosophila melanogaster* adult brain coexpressing *VAChT* (red) and *VGlut* (green) result in Cholinergic/Glutamatergic dual-transmitter neurons (orange). **(E)** Neurons *of Ciona intestinalis* coexpressing *VAChT/CHAT* (red) and *Gad1/2* (green) result in Cholinergic/GABAergic dual-transmitter neurons (yellow). **(F)** Neurons *of Danio rerio* coexpressing *gad1b* (red) and *ChT2* (green) result in GABAergic/Cholinergic dual-transmitter neurons (orange). **(G)** Neurons *of Pogona vitticeps* coexpressing *Gad2* (red) and *SLC17A7* (green) result in GABAergic/Glutamatergic dual-transmitter neurons (orange). **(H)** Neurons *of Mus musculus* (2-3 months of age) coexpressing *Gad1* (red) and *Th* (green) result in GABAergic/Monoaminergic dual-transmitter neurons (yellow). The white arrows indicate some positive cells for coexpression. Threshold: 0.4.

Supplementary Figure 2 *Caenorhabditis elegans* monoaminergic neurons. UMAP plots illustrating the expression of marker genes used to subclassify monoaminergic neurons. TPH, TH and TDC label serotonergic, dopaminergic and tyraminergic/octopaminergic neurons, correspondingly. Black arrows indicate the location of the positive cells for the above-mentioned marker genes in the UMAP plots. TPH: *tryptophan hydroxylase*. TH: *tyrosine hydroxylase*. TDC: *tyrosine decarboxylase*.
