# Supplementary figures and images for: Single-Cell Transcriptomic Reveals Dual and Multi-Transmitter Use in Neurons Across Metazoans
Source: Front Mol Neurosci. 2021 Feb 1;14:623148. doi: 10.3389/fnmol.2021.623148 (PMC7883486; doi:10.3389/fnmol.2021.623148)

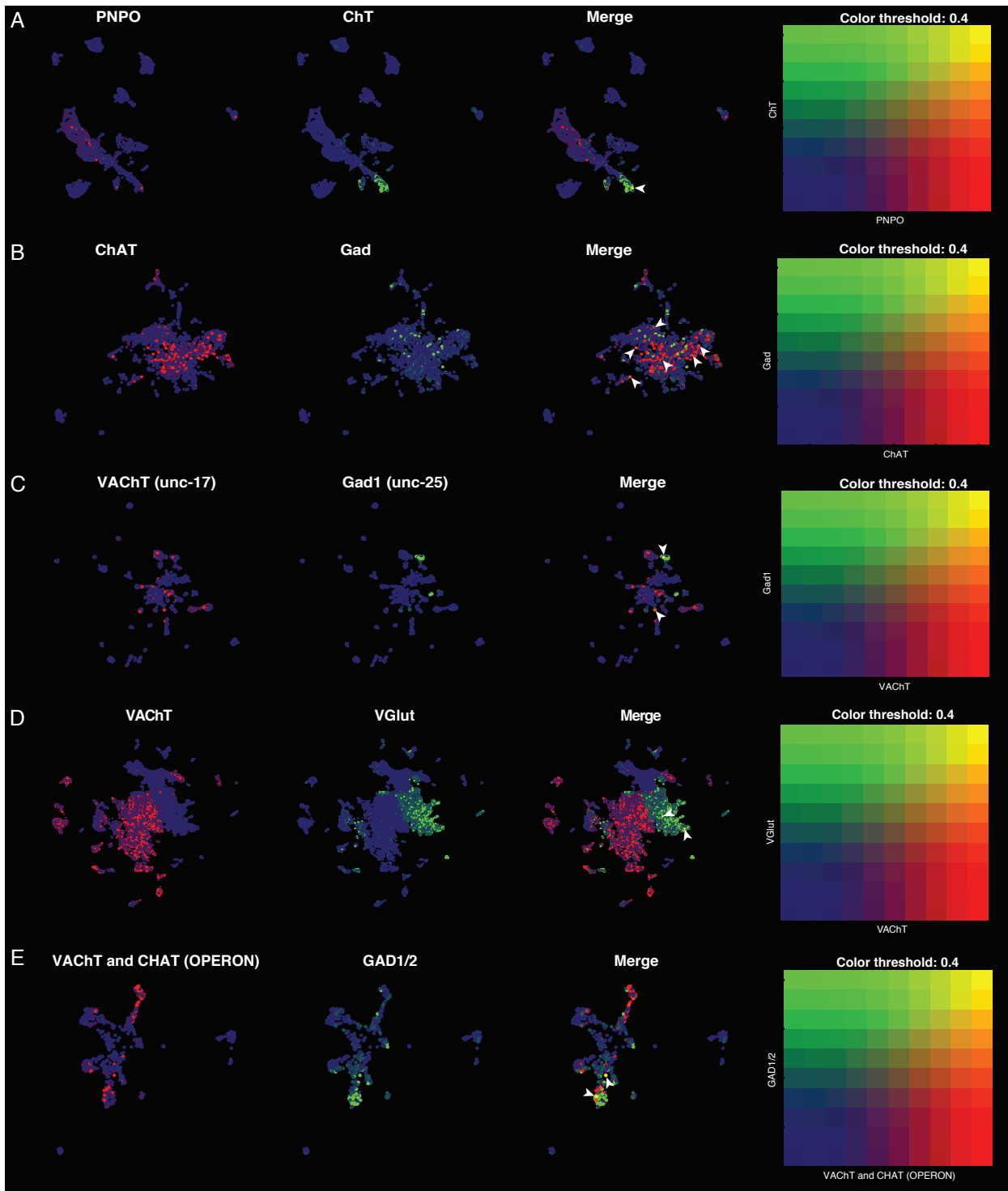

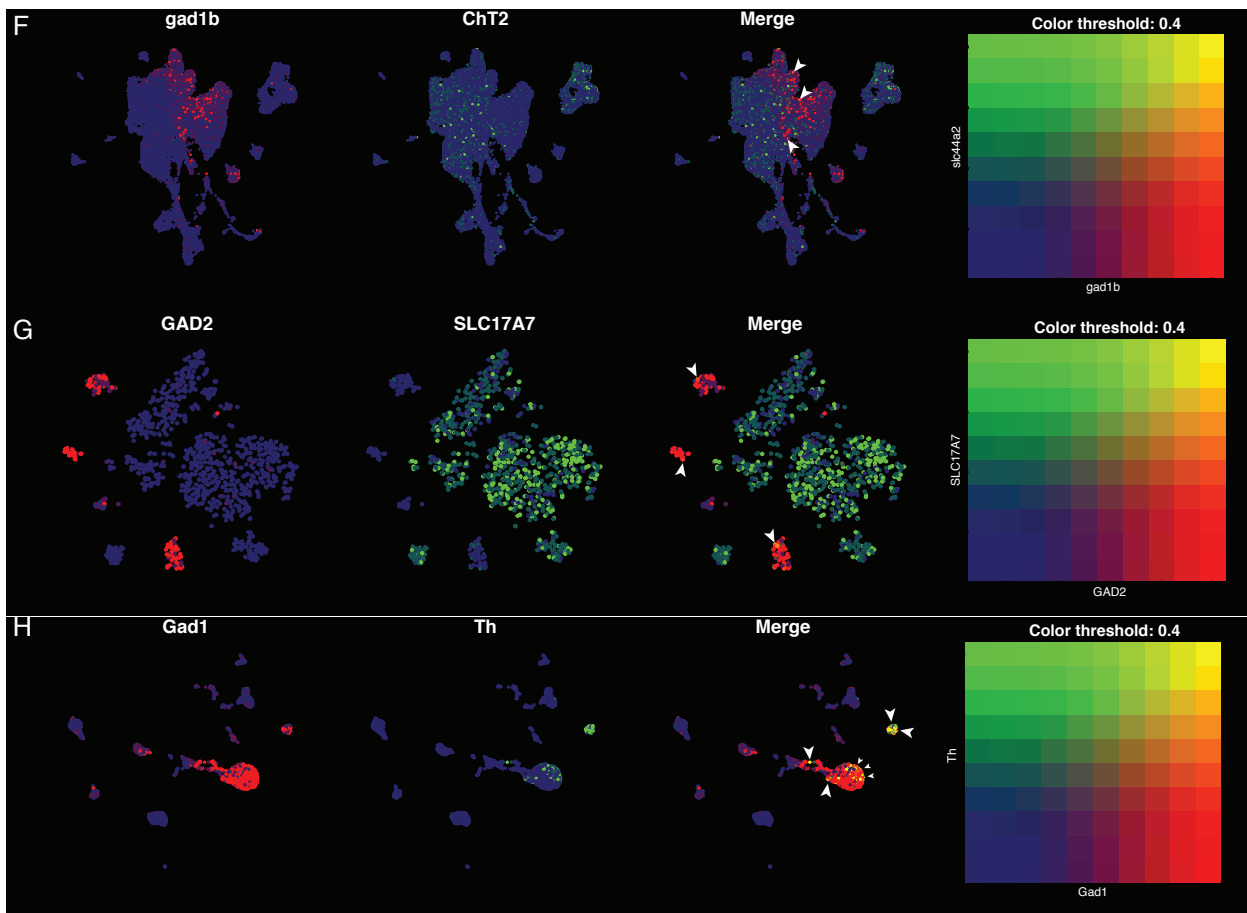

Supplement: Supplementary Figure 1 — Coexpression analysis. UMAP plots illustrating the coexpression of marker genes used to subclassify neurons based on their neurotransmitter phenotypes. (A) Neurons of Hydra vulgaris coexpressing PNPO (red) and ChT (green) result in GABAergic/Cholinergic dual-transmitter neurons (yellow). (B) Neurons of Schmidtea mediterranea coexpressing ChAT (red) and Gad (green) result in Cholinergic/GABAergic dual-transmitter neurons (orange). (C) Neurons of Caenorhabditis elegans coexpressing VAChT (red) and Gad1 (green) result in Cholinergic/GABAergic dual-transmitter neurons (yellow/orange). (D) Neurons of Drosophila melanogaster adult brain coexpressing VAChT (red) and VGlut (green) result in Cholinergic/Glutamatergic dual-transmitter neurons (orange). (E) Neurons of Ciona intestinalis coexpressing VAChT/CHAT (red) and Gad1/2 (green) result in Cholinergic/GABAergic dual-transmitter neurons (yellow). (F) Neurons of Danio rerio coexpressing gad1b (red) and ChT2 (green) result in GABAergic/Cholinergic dual-transmitter neurons (orange). (G) Neurons of Pogona vitticeps coexpressing Gad2 (red) and SLC17A7 (green) result in GABAergic/Glutamatergic dual-transmitter neurons (orange). (H) Neurons of Mus musculus (2-3 months of age) coexpressing Gad1 (red) and Th (green) result in GABAergic/Monoaminergic dual-transmitter neurons (yellow). The white arrows indicate some positive cells for coexpression. Threshold: 0.4. [file Image_1.PDF]
